# The Computational Anatomy of Visual Neglect

**DOI:** 10.1093/cercor/bhx316

**Published:** 2017-11-28

**Authors:** Thomas Parr, Karl J Friston

**Affiliations:** Wellcome Trust Centre for Neuroimaging, Institute of Neurology, University College London, London WC1N 3BG, UK

**Keywords:** active inference, Bayesian, visual neglect, neuropsychology, saccades

## Abstract

Visual neglect is a debilitating neuropsychological phenomenon that has many clinical implications and—in cognitive neuroscience—offers an important lesion deficit model. In this article, we describe a computational model of visual neglect based upon active inference. Our objective is to establish a computational and neurophysiological process theory that can be used to disambiguate among the various causes of this important syndrome; namely, a computational neuropsychology of visual neglect. We introduce a Bayes optimal model based upon Markov decision processes that reproduces the visual searches induced by the line cancellation task (used to characterize visual neglect at the bedside). We then consider 3 distinct ways in which the model could be lesioned to reproduce neuropsychological (visual search) deficits. Crucially, these 3 levels of pathology map nicely onto the neuroanatomy of saccadic eye movements and the systems implicated in visual neglect.

## Introduction

Visual neglect is a common syndrome in which patients neglect one side (typically the left) of space (Halligan and Marshall 1998). It is often caused by right middle cerebral artery strokes, but has also been reported as a consequence of inflammatory (Gilad et al. 2006), metabolic (Auclair et al. 2008), and degenerative (Ho et al. 2003; Andrade et al. 2010) diseases. It has also been observed as a feature of seizure activity (Heilman and Howell 1980; Turtzo et al. 2008; Schomer and Drislane 2015), and as part of a migraine aura (Di Stefano et al. 2013). In addition to the wide range of pathological processes which can cause the syndrome, visual neglect can be caused by a range of anatomical lesions. These include both cortical (Corbetta and Shulman 2002) and subcortical (Karnath et al. 2002) insults. There is some evidence that the heterogeneity of the causes of visual neglect map on to distinct behavioral phenotypes (Hillis et al. 2005; Grimsen et al. 2008; Medina et al. 2009; Verdon et al. 2009), and this has the potential to be exploited clinically and scientifically.

Eye tracking provides one way to characterize behavioral deficits in visual neglect. These measurements have demonstrated that patients with visual neglect perform saccades to the right side of space with a disproportionately high frequency, compared with leftward saccades. This occurs both spontaneously (Fruhmann Berger et al. 2008; Karnath and Rorden 2012) and during search tasks (Husain et al. 2001). While these biases will form the main subject of this article, it is important to note that it may be possible to elicit signs of neglect in patients with no deficit in ocular exploration. For example, in tasks requiring a manual response, it is possible that patients may exhibit a normal pattern of saccadic eye movements, but that they may be impaired in executing a response (Ladavas et al. 1997; Bourgeois et al. 2015). In this article, we consider the control of eye movements, and the conditions that would have to be fulfilled in order to explain the saccadic patterns observed in visual neglect. We aim to show that there is a well-defined and distinct set of conditions that can reproduce the neglect syndrome.

Active inference provides a principled framework in which to define these conditions—in terms of the prior beliefs that a patient would have to possess for their behavior to be Bayes optimal. The notion of optimal pathology might seem a strange one, but the existence of a set of prior beliefs that renders any behavior optimal is mandated by the complete class theorems (Wald 1947; Daunizeau et al. 2010). This means that we can characterize pathology in terms of optimal inference, but in a system or subject that operates under a poor model of its environment (Conant and Ashby 1970). In the following, we briefly review active inference and show how this normative approach can be used to identify the functional lesions that could cause visual neglect. We then propose a neuroanatomical network that is consistent with the neuronal message passing implied by active inference. This allows us to equate functional lesions to anatomical lesions, and to simulate saccadic eye movements for each lesion in silico. We explore the influence of subcortical structures (Karnath et al. 2002) in visual neglect, and the notion that visual neglect is a type of disconnection syndrome (Bartolomeo et al. 2007; He et al. 2007). The article concludes by asking the question whether the different sorts of (saccadic) behavior induced by distinct sorts of lesions this sufficient to identify the locus of the lesion. We address this question using in silico neuropsychology and Bayesian model selection.

The purpose of this paper is to describe the active inference scheme and establish its predictive validity in (simulated) visual neglect. In subsequent papers, we will validate the underlying functional anatomy using eye tracking and MEG in real (normal) subjects. Our ultimate objective is to translate this model into clinical studies—to provide a functionally and biologically grounded characterization of neuronal computations in patients with visual neglect.

## Active Inference

The formal or normative framework used to characterize hemineglect calls on the notion of active inference. Active inference provides a Bayes optimal account of perception and action by appealing to some fundamental (variational) principles that apply to any system that has evolved to maintain an adaptive exchange with its environment. In brief, to sustain their integrity, adaptive systems must minimize the dispersion of their states (Friston et al. 2006). Mathematically, this dispersion corresponds to entropy, and is equivalent to surprise averaged over time (Friston 2009). Surprise, in the information theoretic sense used here, is the negative log probability of making a particular observation. A surprising observation is one that is unlikely under the prior beliefs possessed by an adaptive system, or subject. An intuitive example is that of blood pressure control. Animals are compelled to keep their blood pressure within narrow bounds in order to survive. The baroreceptor system implicitly “expects” blood pressure to be within this range with a high probability, so it is surprising when pressures outside this range are sensed. It is clear from this example that surprise is something to be avoided, if life is to pursue its familiar course. To interact with the environment, without incurring potentially fatal surprises, it is necessary to possess a generative model that describes how observations are generated. In most situations, the processes generating observable outcomes are sufficiently complex that it becomes intractable to compute surprise explicitly. Instead, a quantity called free energy can be computed (Dayan et al. 1995; Beal 2003; Friston 2003). This furnishes an upper bound on surprise, through Jensen’s inequality (The average of a log is less than or equal to the log of an average. This is a consequence of the concavity of the logarithmic function.).

(1) −lnP(o˜)︸Surprise=−ln∑x[P(o˜,x)Q(x)Q(x)]≤−∑xQ(x)ln[P(o˜,x)Q(x)]︸Jensen'sinequlaity=F︸Freeenergy

Minimizing the free energy therefore minimizes surprise (when the bound is tight). In this equation, x represents hidden (latent) variables, and $\tilde{o}$ represents the sequence of observations made over time. The particular form of the hidden variables depends upon the generative model. $P(\tilde{o},x)$ describes the joint probability distribution of observations (i.e., consequences) and hidden variables (i.e., causes) under the generative model. Q(x) is an arbitrary distribution which becomes an approximate posterior as the free energy is minimized. Note that the first equality holds simply because Q(x) on the right hand side can be canceled, resulting in a marginalization of the joint distribution over all hidden variables.

The above demonstrates the (implicit or explicit) free energy minimization in adaptive systems. A system which does not minimize its free energy will fail to bound its entropy and, over time, will cease to exist; that is, will dissipate and decay. Active inference takes this further, by equipping an agent with beliefs about the policy (sequence of actions) it will pursue (Friston, Samothrakis, et al. 2012). A consequence of the imperative to minimize free energy is that, a priori, agents must believe they will minimize the free energy expected and allowable policies. Specifically, policies which are associated with a smaller expected free energy should be considered more likely than those associated with a larger expected free energy.

## Generative Model

### Markov Decision Processes

**Figure 1.**
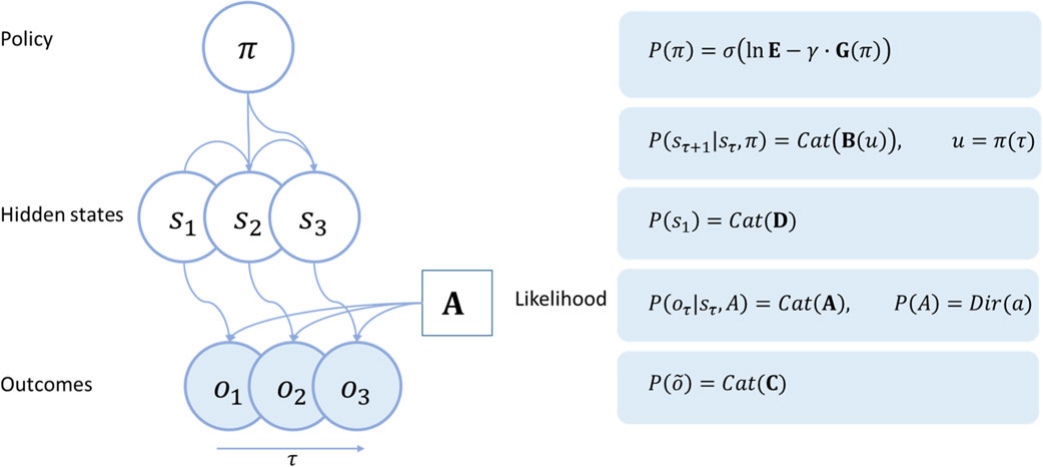
Markov decision process. The Bayesian network shown on the left describes the conditional dependencies in a Markov decision process (MDP). Each variable is shown in a circle, with shaded circles being observed variables. Arrows from one circle to another indicate that the probability of the second variable is conditioned on the first. The forms of these conditional distributions are given in the panel on the right. Cat means a categorical distribution, while Dir is a Dirichlet distribution. σ is a softmax (normalized exponential) function. Please see main text for a fuller explanation of the variables.

Given the discrete, serial, nature of saccadic sampling, an appropriate model structure—for the purposes of this article—is a Markov decision process (MDP) (Mirza et al. 2016). These models are defined in terms of a discrete state space, with observations made at discrete time points. The generic structure of an MDP is shown in Figure 1. The hidden variables in this model are the hidden states, $s_{\tau }$, the parameters of the likelihood mapping, A, and the policy π. The free energy can be expressed in terms of these unknown or hidden quantities (The notation $E_{Q}[\;\cdot \;]$ means the expectation under the distribution *Q*.).

(2) $$F=E_{Q\left(\tilde{s},A,\pi \right)}\left[\ln\;Q(\tilde{s},A,\pi \right)-\;\ln\;P\left(\tilde{o},\tilde{s},A,\pi \right)]$$

An MDP is structured such that observable outcomes depend only on the hidden states. The probabilistic mapping from hidden states to outcomes is expressed in the matrix **A**, in which $A_{ij}=P\left(o_{\tau }=i|s_{\tau }=j\right)$. The hidden states depend only on the previous hidden state, and on the transition matrix **B**, which is a function of the policy. Preferences are specified in terms of the prior beliefs an agent has about the outcomes they will observe, and these are contained in the matrix **C**. **D** determines the probabilities of the initial states. The vectors **E** and **G** correspond to prior expectations about policies and expected free energy respectively (please see below).

**Figure 2.**
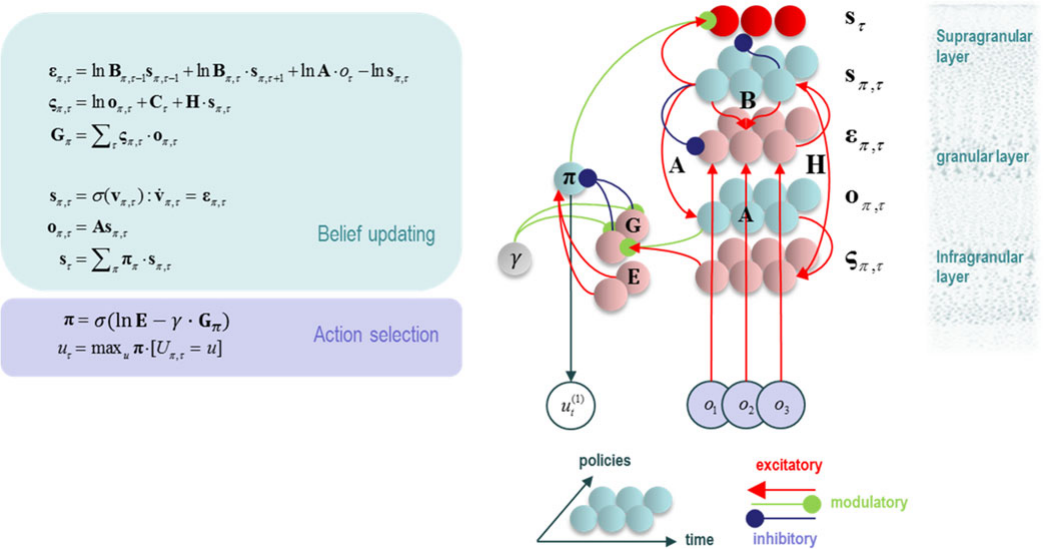
Variational update equations. The equations in the left panel can be obtained through a minimization of the variational free energy with respect to posterior expectations of each variable in the MDP model described in Figure 1. Each expectation is expressed as a vector, each component of which corresponds to the approximate (Q) distribution for a particular value of that variable. H is the entropy of the likelihood matrix. On the right, variables have been assigned to neuronal populations, and the connections between these populations determined from the update equations. The result closely resembles a cortical column with a corticosubcortical loop. The modulation of this loop by the precision (γ) unit, which is thought to correspond to dopaminergic activity (Friston et al. 2013) is suggestive of a nigrostriatal influence. The 2 opposing inputs to the policy unit could reflect the direct and indirect basal ganglia pathways. Notably, E (prior expectations about policies) does not depend on current beliefs about states, while G (expected free energy) is context dependent. Correspondingly, indirect pathway neurons in the striatum have relatively small dendritic trees with limited cortical input, while direct pathway neurons have very large dendritic trees (Gertler et al. 2008).

This generative model allows the factorization of $P(\tilde{o},\tilde{s},A,\pi )$ into conditionally independent factors. Using a “mean field approximation,” we can additionally factorize $Q(\tilde{s},A,\pi )$ into approximately independent factors. It is then possible to derive update equations for each factor of Q, by taking the derivative of the (variational) free energy with respect to that factor, and setting the result to zero (see Appendix for the derivation of the hidden state updates). In doing so, the update equations shown in Figure 2 are obtained (Friston, FitzGerald, Rigoli, Schwartenbeck and Pezzulo 2016). Reassuringly, when the variables in these equations are mapped out in terms of the influence each has over the others, the emergent structure closely resembles the architecture of cortical microcircuits, cortical hierarchies, and even corticosubcortical loops involved in policy evaluation. This loop is consistent with the structure and function of the basal ganglia (Jahanshahi et al. 2015). See Figure 2.

### Memory and Short-Term Plasticity

Given that the probability distributions are specified as categorical distributions, the appropriate conjugate prior for the likelihood A matrix is a Dirichlet distribution. This means that the probability can be represented simply in terms of Dirichlet concentration parameters. For each state, $s_{\tau }=j$, there are a set of Dirichlet parameters, $a_{ij}$, one for each outcome, $o_{\tau }=i$, which could be associated with this state. These are initially “pseudo-observations,” as no observation has yet been made. The belief about the probability of outcome i given state j is as follows:

(3) $$A_{ij}=\frac{a_{ij}}{\sum_{k}a_{kj}}$$

As observations are made, the agent is able to learn—that is, accumulate its Dirichlet parameters—to better fit its observations. This process of learning simply involves increasing the Dirichlet parameter representing a particular outcome when that is observed (Beal 2003; Blei et al. 2003). The amount it is increased by the (approximate) posterior probability that each hidden state was occupied when the observation was made. This allows an agent to remember the observations they sampled when they believed they were in a particular state (Friston, FitzGerald, Rigoli, Schwartenbeck, O’Doherty, et al. 2016). The notion that the mapping between representations of 2 variables should be increased when the 2 are simultaneously active is strikingly similar to Hebbian plasticity (Hebb 1949; Brown et al. 2009). This analysis suggests that this form of memory could be implemented by short-term changes in synaptic efficacy.

An important consequence of the Dirichlet parameterization concerns the scaling of parameters. The scaling of the Dirichlet parameters does not influence the values in the likelihood matrix. However, it does influence the degree to which these change following an observation. If all the concentration parameters are very large (as would be the case if many past observations had been made), a single observation will make a very small difference to the likelihood, A. If the parameters are very small, an observation can trigger one-shot learning, suggesting a rapid short-term plasticity effect. Such effects have been proposed as one mechanism underlying working memory (Mongillo et al. 2008). This behavior is of particular interest in the current context, as will become apparent in the next section, where the form of the MDP used to model hemineglect is described.

## Saccadic Cancellation Task

**Figure 3.**
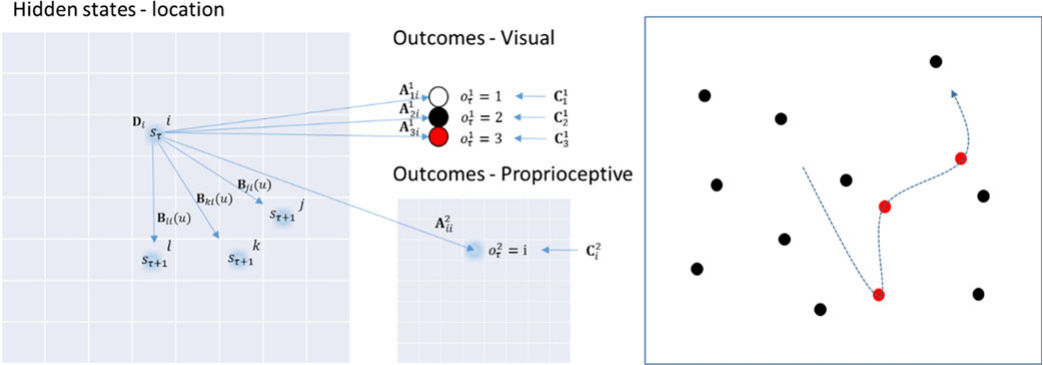
Generative model for the saccadic cancellation task. The structure of the particular generative model used for the saccadic cancellation task is shown on the left. See Fig. 1 for the definitions of the symbols used. The hidden states in this model are the locations in the visual field that are fixated. These are indicated by the 8 × 8 grid on the left of this figure. The start location, is specified by **D**. The agent may saccade to any location on the grid (three possible saccades are shown), and the particular saccade is defined by u, which selects the appropriate B matrix. Each component of this matrix defines the probability of a saccade to a given location (j,k, or l in the figure), given a current location (i in the figure). There are 2 A matrices which provide a probabilistic mapping from the hidden states to the visual ($A^{1}$) or proprioceptive ($A^{2}$) outcome modalities. Prior preferences are defined by the C matrices, which are defined for each modality. On the right is a depiction of the structure of the task resulting from the generative model. The dotted line is the saccade path, and this demonstrates the change from black to red of targets as they are canceled.

The task performed by the particular MDP model used in this work is based on the pen-and-paper line cancellation task (Albert 1973; Fullerton et al. 1986). This task is used to assess visual neglect clinically, and is very sensitive (Ferber and Karnath 2001). Despite its popularity, it is worth noting that there are many possible reasons that performance of this task might be impaired. We will demonstrate this for a few of these reasons below. We will use a saccadic version, which involves presenting the subject with an array of targets that can be placed at various locations on an 8 × 8 grid. The task is to look at each of the targets until all targets have been sampled (i.e., canceled). When a target has been fixated, it changes color from black to red (see the right panel in Fig. 3), indicating that it has been seen. The model used to emulate this behavior is shown in Figure 3, in terms of the variables in the MDP. The only hidden states in this model correspond to the location currently foveated. An identity matrix maps these deterministically to proprioceptive observations, ensuring there is no uncertainty about the hidden state (i.e., where the subject is currently looking). The uncertainty in the model is contained in the (likelihood) mapping from hidden states to visual outcomes. There are 3 possible outcomes: no target (white), target (black), and canceled target (red). The prior preferences of the simulated agent are that it has equal preferences over all proprioceptive outcomes, prefers to see targets that have not been canceled, and does not expect to see targets that have already been canceled. The subject begins with (almost) uniform beliefs about the A matrix (i.e., what will happen if she looks at a particular location). However, these incorporate very weak, but accurate, beliefs concerning the locations of the targets. On foveating a target, the first visual outcome is a black target. This observation allows the appropriate Dirichlet parameters to be accumulated. During fixation, the target changes from black to red, and this causes further changes in the Dirichlet parameters, so that the subject remembers she has already canceled that location. This implements a synaptic form of spatial working memory (Mongillo *et al.* 2008). The subject may saccade to any location at any time, meaning there are 64 possible actions, each of which is associated with a corresponding transition (**B**) matrix. Having established the basic form of the generative model, sufficient to simulate visual search, we now turn to the finer details of the implicit epistemic foraging and how salient targets are selected—and what can go wrong under pathological priors.

## Computational Neuropsychology

In principle (under the complete class theorem), all neuropsychological syndromes can be formulated in terms of active inference. The challenge is to find the prior beliefs a subject would have to possess to render their behavior Bayes optimal. For visual neglect, we consider the abnormal patterns of saccadic eye movements in patients (Husain *et al.* 2001; Fruhmann Berger *et al.* 2008; Bays et al. 2010; Karnath and Rorden 2012), and the beliefs which would engender these patterns. For each saccadic policy, the generative model specifies the prior probability that the policy will be pursued. By analysing the form of this prior belief, one can develop a differential diagnosis for the computational lesions in visual neglect. As noted above, the prior belief about policy should depend on the expected free energy. The smaller the expected free energy under a policy, the more likely it will be pursued. We can express this formally as follows:

(4) P(π)=σ(−γ⋅G(π))

In this equation, σ(⋅) is a softmax function, which ensures the resulting distribution will sum to one, making it a proper probability distribution. The scale parameter γ is an inverse temperature parameter, which acts as a precision over policy priors. G(π) is the expected free energy associated with each policy π. This is defined as the sum of expected free energies for each future time point.

(5) $$G\left(\pi \right)=\underset{\tau >t}{\sum }G(\pi ,\tau )$$

The expected free energy has a similar form to that of the variational free energy. However, there are 2 key differences. The first is that it must be conditioned on the policy pursued, and the second is that, by definition, future observations have not yet been made. This means the expectation in Equation 2 must now include beliefs about future outcomes. Defining $\tilde{Q}\left({ο}_{\tau },s_{\tau },A|\pi \right)=Q\left(s_{\tau },A|\pi \right)P\left(o_{\tau }|s_{\tau },A\right)$ allows us to express expected free energy as follows:

(6) $$G\left(\pi ,\tau \right)=E_{\tilde{Q}}\left[\ln\;Q\left(s_{\tau },A|\pi \right)-\;\ln\;P\left(o_{\tau },s_{\tau },A|\pi \right)\right]$$

Rearranging this, we can separate out the key terms that influence policy selection in the generative model described above.

(7) $$\begin{array}{c} G\left(\pi ,\tau \right)=\underset{\mathrm{Expected cost}}{\underset{︸}{-E_{\tilde{Q}}\left[\ln\;P\left(o_{\tau }\right)\right]}}\\ \;-\;E_{Q\left(o_{\tau }|\pi \right)}\underset{\mathrm{Salience}}{\underset{︸}{[D_{\mathrm{KL}}\left[P\left(s_{\tau }|o_{\tau },\pi \right)||Q\left(s_{\tau }|\pi \right)\right]}}\\ \;+\;D_{\mathrm{KL}}\underset{\mathrm{Novelty}}{\underset{︸}{\left[P\left(A|o_{\tau },\pi \right)||Q\left(A|\pi \right)\right]]}} \end{array}$$

The second (salience) term in this equation, in the context of the generative model used here, is identical for all policies. This is because the identity mapping from the hidden states representing locations to the proprioceptive outcomes allows the subject to infer location in visual space with no certainty. This means there is no information gain or epistemic value that would otherwise resolve uncertainty about the hidden state. The key terms that determine policy selection are the first and third. The former implies that a policy which is expected to fulfill the agent’s prior beliefs (preferences) about outcomes has a lower expected free energy than one which does not. The latter suggests that a policy which affords the greatest change in the beliefs about the likelihood mapping, $\;P\left(A|o_{\tau },\pi \right)$, from beliefs prior to seeing counterfactual outcomes, Q(A|π), has the lowest expected free energy. Heuristically, policies that elicit observations that enable large Bayesian belief updates become more attractive. In other words, the subject will be attracted to novel contingencies that resolve uncertainty about the consequences of being in a particular state; that is, the likelihood mapping.

In short, prior preferences and novelty are both important factors in determining the selection of a location to saccade to. This implies 2 possible computational mechanisms for visual neglect. A subject may have a prior belief that she will experience the proprioceptive outcomes corresponding to the right side of space with a greater probability than those corresponding to the left. Alternatively, the subject may be more confident in her beliefs about the mapping from states to outcomes on the left, and therefore consider the right side of visual space novel. This is equivalent to starting with very large Dirichlet parameters (corresponding to a large number of pseudo-observations) for locations on the left. This follows because an observation resulting from a saccade to the left will induce a small change in beliefs about the likelihood mapping.

A third possibility relates to (baseline) prior beliefs about policies that may not depend upon expected free energy. Although active inference mandates that an agent believes it will pursue policies which minimize its expected free energy, it does not preclude fixed prior beliefs over policies which, in visual neglect, might identify saccades to the right to be a priori more likely than those to the left. To express this formally, we can augment the expression for priors over policies as follows:

(8) lnP(π)=lnE−γ⋅G(π)

Here, **E** expresses the prior beliefs about policies that do not depend on the expected free energy. In this form, the (log) priors over policies are expressed as a linear function of expected free energy, where **E** corresponds to the *y*-intercept and precision is the sensitivity or slope.

In summary, the above formal considerations have led us to identify 3 possible synthetic lesions which could give rise to visual neglect. These are changes in the priors over policy **E**, the Dirichlet parameters of the beliefs about $A^{1}$, and the priors concerning proprioceptive outcomes, contained in the matrix $C^{2}$. In the following section, we review plausible neurobiological substrates for each of these computational pathologies.

## The Neuroanatomy of Hemineglect

### The Dorsal Attention Network

The superior colliculus, in the midbrain, is a key site for the control of saccadic eye movements (Raybourn and Keller 1977). It is also a point of convergence for the cortical and subcortical structures involved in oculomotor control (Künzle and Akert 1977; Berson and McIlwain 1983; Fries 1984, 1985; Shook et al. 1990; Gaymard et al. 2003). The substantia nigra pars reticulata, a GABAergic output nucleus of the basal ganglia, projects directly to the colliculus (Hikosaka and Wurtz 1983), as do cortical areas including the frontal eye fields (Künzle and Akert 1977) and the lateral intraparietal cortex (Gaymard *et al.* 2003) (sometimes called the parietal eye fields (Shipp 2004)). These dorsal frontal and parietal areas constitute the dorsal attention network (Corbetta and Shulman 2002), and communicate via the first branch of the superior longitudinal fasciculus (Makris et al. 2004; Bartolomeo et al. 2012). The frontal eye fields are well placed to house the hidden states representing eye position, while dorsal parietal areas are suited to the representation of proprioceptive information. The former are known to contain spatial maps in egocentric space, as evidenced by demonstrations that stimulation of neurons in this region induce saccades that end in specific egocentric eye positions (Bruce et al. 1985; Sajad et al. 2015). The latter contain neurons that are modulated by multiple spatial reference frames (Andersen et al. 1985; Pouget and Sejnowski 2001).

The parietal cortex is part of the dorsal visual stream, thought to carry information about the location of a stimulus (Goodale and Milner 1992; Ungerleider and Haxby 1994). In the present context, the first branch of the superior longitudinal fasciculus would perform a coordinate transformation, bringing spatial information about a stimulus into egocentric coordinates; suitable for planning eye movements. This suggests that the superior longitudinal fasciculus corresponds to the connectivity or mapping encoding the likelihood matrix $A^{2}$ (Fig. 3). In our model, this is an identity mapping, but this is only the case when the head is assumed to be in a fixed position. A model which allowed for head movements would require this matrix to represent a more complex coordinate transform. Given proprioceptive outcomes are represented in the dorsal parietal regions; inputs to this region must represent prior beliefs concerning proprioception. A candidate structure providing this information is the pulvinar, which is involved in visual search behaviors (Ungerleider and Christensen 1979). The connections from this region would then encode the $C^{2}$ matrix.

### The Ventral Attention Network

Despite the important role of dorsal frontoparietal areas in the generation of saccadic movements (Corbetta et al. 1998), it is more ventral frontoparietal lesions which are associated with the visual neglect syndrome (Corbetta et al. 2000; Corbetta and Shulman 2002, 2011). These regions are the constituents of the ventral attention network, and are connected by the third branch of the superior longitudinal fasciculus (Rushworth et al. 2005; Bartolomeo *et al.* 2012). The parietal part of this network includes areas in the region of the temporoparietal junction, closer to the temporal regions associated with the ventral visual stream. This component of the visual system has been described as the “what” pathway (Ungerleider and Haxby 1994), propagating information concerning stimulus identity to complement the “where” information of the dorsal stream. The ventral temporoparietal regions are then good candidates for the representation of the visual outcome modality of the model, allowing them to influence eye movements in a stimulus-driven manner (Shomstein et al. 2010). Connections from the ventral frontal cortex could then carry information concerning prior beliefs (equivalent here to the instructions a subject would be given), consistent with the proposed role of areas in this region in representing task demands (Duncan 2001; Dosenbach et al. 2006) and in target detection (Stevens et al. 2005). This suggests that the third branch of the superior longitudinal fasciculus is the anatomical substrate of $C^{1}$.

Notably, the ventral attention network is lateralised to the right cerebral cortex, while the dorsal network is much more symmetrical (Corbetta et al. 2002; Thiebaut de Schotten et al. 2011; Vossel et al. 2012). This is consistent with the notion that temporal regions could represent the “what” modality, as identity is largely independent of location, and therefore does not require a bilateral representation (Parr and Friston 2017). There is evidence to suggest that this unilateral representation of identity is right lateralised (Warrington and James 1967, 1988; Warrington and Taylor 1973), while left sided homologues relate to object naming (Kirshner 2003). We note that, although temporoparietal regions are thought to play a role in target detection (Corbetta *et al.* 2000), they do not appear to be necessary for object recognition. The involvement of the ventral network is consistent with the fact that visual neglect is frequently associated with right hemispheric lesions.

This leaves the question of how lesions in ventral regions produce the saccadic deficits that might be expected from dysfunction of areas which are directly involved in saccadic control. One answer to this question is that visual neglect involves dysfunction of the dorsal network as a consequence of the failure of the ventral network, or of the interaction of the 2 networks (He *et al.* 2007). The 2 networks are joined by the second branch of the superior longitudinal fasciculus (Thiebaut de Schotten *et al.* 2011), and it has been proposed that visual neglect represents a functional disconnection syndrome involving this pathway. Given that this branch connects the parietal part of the ventral system to the frontal part of the dorsal system, this corresponds exactly to the mapping described by $A^{1}$. It is interesting that this tract, heavily implicated in visual neglect (Doricchi and Tomaiuolo 2003; Thiebaut de Schotten et al. 2005), appears to be the anatomical homologue of the mathematical entity identified above as a candidate for pathological priors—on purely theoretical grounds.

### Subcortical Structures

As stated above, an important input to the superior colliculus is the substantia nigra pars reticulata. This structure is a point of convergence for the direct and indirect pathways through the basal ganglia. Both of these originate from the striatum, which comprises the caudate nucleus and putamen. In visual neglect patients with subcortical lesions, there is substantial lesion overlap found in the putamen, and to a lesser degree in the caudate (Karnath *et al.* 2002). As indicated in Figure 2, the putamen is involved in the evaluation of policies. This fits with the proposed role of the basal ganglia. Additionally, as policies that are independent of the expected free energy are equivalent to habitual behavior, it makes intuitive sense that pathological biasing of policies would take place within a structure which is involved in habit formation; that is, the striatum (Yin and Knowlton 2006). The consistency of the anatomy of the basal ganglia with the policy update equations is further enhanced when the hierarchical extension of these equations is considered (Friston et al. 2017). These imply multiple parallel loops, originating and ending in the cortex, closely resembling those described in subcortical structures (Haber 2003).

The pulvinar is another subcortical region that is strongly implicated in visual search and neglect—and, as mentioned above, connects to dorsal parietal areas (Weller et al. 2002; Behrens et al. 2003). This makes it a plausible anatomical substrate for the representation of prior beliefs about proprioceptive outcomes. This is consistent with accounts of the pulvinar in directing attention (Shipp 2003; Kanai et al. 2015) and eye movements (Petersen et al. 1985), and as a “salience map” (Robinson and Petersen 1992 ; Veale et al. 2017).

There are other possible lesions which could be accommodated by this model. For example, unilateral disruptions of the connections from the substantia nigra pars reticulata to the superior colliculus (Schiller et al. 1980, 1987; Hikosaka and Wurtz 1985a), or of the dopaminergic modulation of the striatum (Kato et al. 1995; Kori et al. 1995), have been shown to cause visual neglect-like syndromes. However, these lesions are rarely reported as causes of neglect in human patients. We have prioritized the lesions corresponding to the white matter tract that connects the dorsal and ventral attention networks, in addition to 2 common subcortical lesions; the putamen and pulvinar. These closely resemble the theoretically motivated lesions of $A^{1}$, E, and $C^{2}$.

### Interhemispheric Interactions

**Figure 4.**
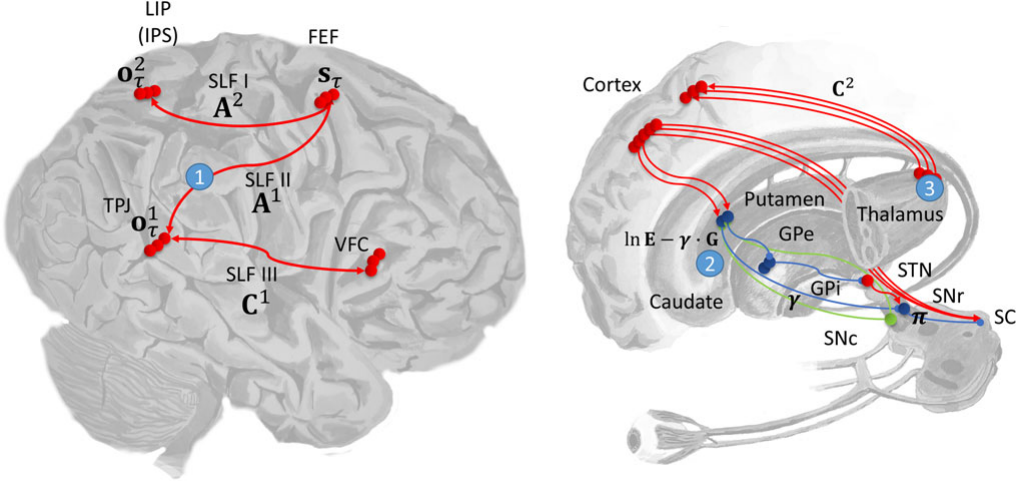
Computational anatomy and lesion sites. This schematic illustrates the proposed mapping from the computational entities implicated by the model (Figs 2 and 3) and their neuroanatomical substrates. On the left the dorsal and ventral attention networks are shown. The former involves the frontal eye fields (FEF) and posterior parietal areas in the region of the lateral intraparietal area (LIP) and intraparietal sulcus (IPS). The frontal areas of this network are assumed to represent the hidden states, corresponding to the current fixation location. The parietal component represents proprioceptive outcomes (eye position). The connection between these frontoparietal areas is the first branch of the superior longitudinal fasciculus (SLF I), mediating the likelihood mapping between the hidden states and proprioceptive outcomes ($A^{2}$). The ventral attention network includes the ventral frontal cortex (VFC) and the temporoparietal junction (TPJ). These are connected by SLF III, which could carry prior preferences about visual outcomes ($C^{1}$). Visual outcomes are assumed to be represented in the TPJ, which suggests the SLF II is the mapping from hidden states to visual outcomes ($A^{1}$), and it is in these connections that the beliefs about the target locations are encoded. Prior preferences for proprioceptive outcomes are assigned to the pulvinar, a nucleus of the thalamus. On the right the connections from the pulvinar to the dorsal parietal cortex (LIP) are shown. These are portrayed as conveying expectations about (proprioceptive) outcomes in $C^{2}$. In addition, the pathways through the basal ganglia are also shown. The policy evaluation processes shown in Figure 2 are depicted as stages in the direct pathway. In this scheme, the putamen evaluates the expected free energy, and baseline policy priors, E. These are modulated by dopaminergic inputs from the substantia nigra pars compacta , in proportion to their precision γ, and the output of the putamen is transformed by the substantia nigra pars reticulata into a distribution over policies. The simulated lesions we considered are numbered: 1—SLF II; 2—Putamen; 3—Pulvinar. As in the previous figure, red connections are excitatory, blue inhibitory, and green modulatory.

In the above, our focus has been on disruption of the communication between posterior and frontal cortices, and on subcortical disconnections within the right hemisphere. Importantly, there is good evidence (Vuilleumier et al. 1996; Rushmore et al. 2006; Dietz et al. 2014) that neglect involves interhemispheric imbalances in addition to intrahemispheric disruptions (Bartolomeo *et al.* 2007; Bartolomeo 2014). This is a key feature of an existing model of neglect (Kinsbourne 1970). Fortunately for our framework, the 2 are inherently linked. Examination of the equations in Figure 2 (and the Appendix) reveals 2 key features in the belief updates for hidden states. The first feature is that beliefs about states are conditionally dependent upon policies. This means that any bias towards policies favouring saccades to the right will increase the probability, on taking a Bayesian model average over policies, of a fixation location on the right. Given the contralateral cortical control of eye movements, this corresponds to increased left hemispheric activity. The second important computational feature is the softmax function, which ensures posterior beliefs over allowable fixation locations sum to one (i.e., ensures a proper probability distribution). Such a constraint could be biologically implemented by inhibitory interactions within and between the 2 frontal eye fields. In other words, if fixations on the right side of space are considered more probable, it must be the case that leftward fixations are less probable. This necessarily implements a form of interhemispheric competition—a competition that is won by the left hemisphere if any of the lesions described in the previous section bias policies towards rightward saccades (Fig. 4).

## Simulating Hemineglect

### Heterogeneous Pathology to Homogenous Syndrome

**Figure 5.**
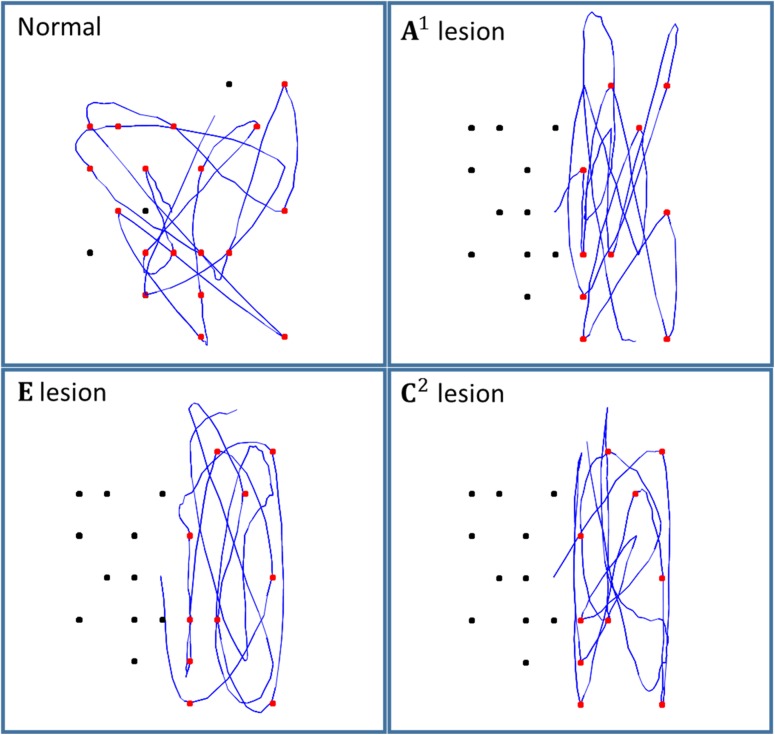
Simulated saccadic cancellation task. Each of the panels shows the simulated eye tracking data (blue) during 20 saccades. In all cases, the target array was the same. The upper left panel shows the performance of the model with no simulated lesions. The upper right panel shows the results when the $A^{1}$ Dirichlet parameters were increased for the left hemifield, corresponding to a functional disconnection of the second branch of the right superior longitudinal fasciculus. The lower left panel shows performance when there is a biasing of policy selection, simulating a lesion of the putamen. The lower right panel represents a lesion of the prior beliefs about proprioceptive outcomes, which relates to a deficit in the inputs to the dorsal parietal cortex, likely from the pulvinar.

Figure 5 shows the results of running the simulation for 20 saccades, under different prior beliefs (i.e., lesions). Strikingly, all 3 lesioned models produce very similar behavioral patterns. This heterogeneity of functional lesions is consistent with the diverse set of anatomical lesions known to cause visual neglect. While the nonlesioned model samples both sides of space, all 3 lesions cause a bias towards sampling the right side of space. This biased sampling is very similar to that observed in visual neglect patients (Bays *et al.* 2010). It is worth noting that people may have additional priors over their policies (contained in E), that result in a slightly different pattern of saccadic search than that depicted in Figure 5. For example, people might have a prior bias towards performing a saccade to a nearby target. We have omitted this additional prior, as our aim is to present a minimal model that reproduces the important features of neglect.

The functional disconnection induced by altering the Dirichlet parameters of $A^{1}$ effectively increases the novelty associated with saccades to the right hemifield. This corresponds to the functional disconnection of the dorsal and ventral attention networks, and can be thought of as impairing the “capture” of attention by salient stimuli, consistent with existing theories of visual neglect (Ptak and Schnider 2010) and attention (Shulman et al. 2009). The simulated pulvinar lesion causes the agent to fulfill their prior beliefs that they are more likely to be looking at the right side of space, and the lesioned putamen biases policy selection in favor of saccades in this direction.

While mechanistically distinct, the behavioral profiles of each of these lesions do not appear to lend themselves to precise diagnoses in terms of observable behavior. In the next subsection, we consider a more realistic approach to spatial representations. We follow this with an attempt to determine whether the syndrome generated by these lesions is really as homogenous as it appears, or whether it is possible to identify the lesion from saccadic behavior.

### Representing Visual Space

Spatial representations in the brain involve multiple segregated spatial scales, and resolutions. The magnocellular system, for example, carries information with a relatively low spatial resolution, while the parvocellular system provides higher resolution information (Livingstone and Hubel 1988; Zeki and Shipp 1988, 1989; Nealey and Maunsell 1994). Visual neglect provides further evidence for the brain’s use of multiscale spatial representations. One example of this is the Ota search task (Ota et al. 2001) in which participants are asked to identify, from an array of shapes, which shapes are complete. While some visual neglect patients fail to address any of the left hand side of the array (“egocentric visual neglect”), others address all shapes, but are impaired in determining which shapes are complete (“allocentric visual neglect”). Specifically, those shapes which have a deficiency on their right side are correctly identified as incomplete, while those deficient on their left side are incorrectly identified as complete.

While many accounts have described the 2 perceptual deficits in terms of different spatial reference frames (Medina *et al.* 2009), it has been argued that both forms are actually different manifestations of an egocentric visual neglect (Driver and Pouget 2000; Corbetta and Shulman 2011). If both are considered to take place in the same reference frame, the 2 behavioral patterns would be consistent with visual neglect operating at a coarse spatial scale in the first case, and a finer scale in the second.

Equipping the model with a multiscale representation is simple to do in our generative model; instead of representing each of the 64 locations at a high resolution, we can encode each location using 3 levels (i.e., factors) of resolution, each level divided into 4 quadrants that, collectively, specify 4^3^ = 64 locations. Technically, this means the **A** matrix now becomes 3 matrices encoding the likelihood mappings at low, intermediate, and high levels of resolution. Functionally, this means that the subject perceives visual input at 3 levels of resolution—and can entertain uncertainty (and novelty) at any level. This also means we have the opportunity to model pathological (prior) biases at the level of quadrants of the visual field, quadrants within each quadrant and quadrants within those quadrants.

**Figure 6.**
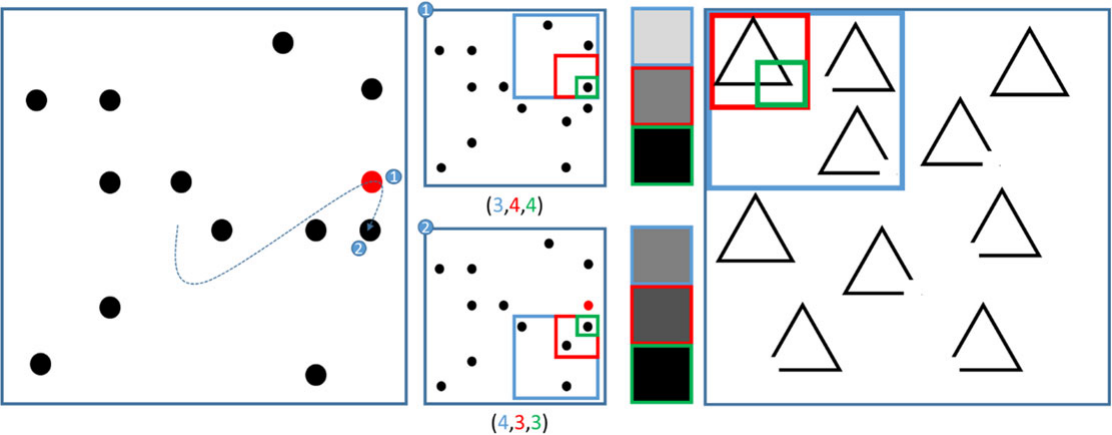
Multiscale representations of space. In the illustration on the left, 2 fixation points in a sequence of saccades are highlighted. This is to demonstrate their representation in terms of a multiscale spatial state space. In the center left, this state space is shown for each fixation point. This specifies a location in an 8 × 8 space, as before. However, the location is specified in terms of which quadrant (blue), which subquadrant (red) and which subsubquadrant (green) the location is found. These 3 specifications constitute the hidden states of the multiscale model. An advantage of this model is that it allows visual outcomes to be defined at different resolutions. This is shown in the center right. Each outcome corresponds to the density of targets in the quadrant, subquadrant, and subsubquadrant currently fixated. Darker shades indicate a greater density. Note that the finest resolution is at the level of individual locations, so density is equivalent to the presence or absence of a target. Canceled targets appear red at this level only—lower resolutions are considered to be color-blind; consistent with the properties of the magnocellular system (Hubel and Livingstone 1987). As a saccade is made from a quadrant containing 3 targets to one containing 4, the lowest resolution (blue frame) outcome becomes denser. Similarly, the subquadrant representation (red frame) becomes darker, as a subquadrant containing only one target is followed by a subquadrant containing 2. The finest resolution (green frame) represents the maximum density (one target) for both fixation locations. The illustration on the right motivates the multiscale representation in terms of the Ota task. This shows one quadrant of an array of shapes. If the blue frame was biased towards occupying the right side of the array, this would resemble an egocentric hemineglect. If the green frame were biased towards the right, this would be closer to an allocentric hemineglect.

**Figure 7.**
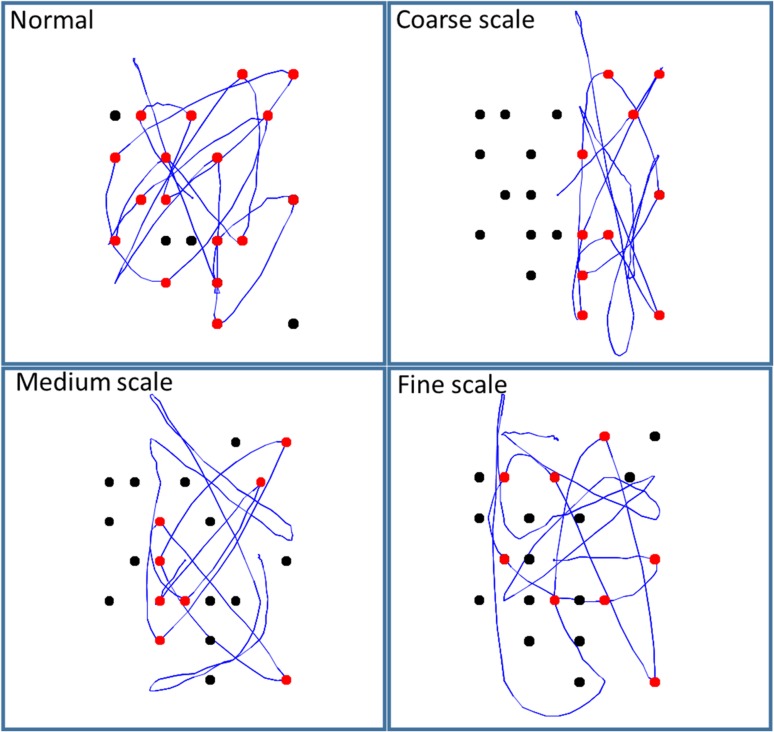
Lesions at different spatial scales. By changing the number of the initial Dirichlet parameters, we have simulated hemineglect at 3 resolutions. As can be seen in the above, the course scale representation biases saccades to the right side of the array, similar to the patterns seen in Figure 5. The medium scale representation biases saccades to the right side within each of the 4 quadrants of visual space. Hemineglect at the finest scale biases saccades to the right of each subquadrant (comprising 4 possible locations). For larger targets, but the same spatial scales, each of these biased sampling policies would produce results very similar to those observed in patients performing the Ota task.

Figure 6 shows a multiscale representation in our model, and its application to the saccadic cancellation task. The right panel shows how the Ota task was used to motivate this approach. If visual neglect is induced at a coarse scale—that is, quadrant enclosed by the blue frame—an egocentric behavioral pattern of saccadic sampling would be expected. However, if induced at a finer scale (green frame), neglect would cause an allocentric pattern. Figure 7 shows the simulated eye tracking data generated under this multiscale representation. Lesions are shown at each spatial scale and are induced by scaling the corresponding Dirichlet concentration parameters. The other 2 types of functional lesion produce similar results. Crucially, different spatial scales of visual neglect could reflect different lesion topologies, as more ventral lesions have been associated more with neglect at the object scale (Grimsen *et al.* 2008; Medina *et al.* 2009; Verdon *et al.* 2009).

A simplification we have made in the generative model we have used is that we have assumed the head position is stationary. This allows us to treat the coordinate transform, performed by the first branch of the superior longitudinal fasciculus, as an identity transformation. If we did not make this assumption, the transformation would have to be modulated by a set of hidden states representing the head position, as in established models of parietal contributions to attention (Pouget and Sejnowski 1997, 2001). The influence of head position over the reference frame—in which neglect is induced—allows for the possibility of different egocentric coordinate systems. However, it may be that a set of egocentric reference frames are insufficient, on their own, to explain some neglect phenomena. There is evidence that the orientation of the axes of reference frames can be influenced by the spatial configuration of visual stimuli (Driver et al. 1994; Li et al. 2014), but the inferences involved in these processes lie outside the scope of this article. Importantly, deficits that are classically described as “object-centered” are rarely seen in the absence of “egocentric” deficits (Rorden et al. 2012; Yue et al. 2012). This suggests that such deficits are not an essential part of the neglect syndrome, but may occur in larger lesions that compromise additional connections.

In summary, we have seen that a normative (active inference) model of visual searches and biased (visual) sampling can provide a sufficient, if minimal, account of the functional deficits observed in patients during line cancellation tasks. The computational architecture and message passing implied by the active inference scheme is remarkably consistent with the known functional anatomy of visual search and saccadic eye movements—and the deficits in epistemic foraging seen in patients with neglect. In the final section, we turn to the practical issues of using this sort of model to make inferences about lesions on the basis of saccadic eye movements.

## Computational Lesion Deficit Analysis

We have established that, just as with anatomical lesions, there are several functional lesions that can induce very similar behavior. This raises an important question. Is the mapping from lesion to behavior truly a many-to-one mapping? In other words, is it possible, given the (simulated) behavioral data, to determine which lesion model generated it? If so, this could have important implications for clinical diagnosis, as it would allow the separation of distinct functional categories of visual neglect.

To answer this question, we used synthetic eye tracking data from each of the lesion models. To assess the ability of the paradigm to disambiguate among lesions, we computed the log likelihood of simulated behavior for every combination of lesion and model. This log likelihood or evidence was computed by summing the likelihood of each saccade under the posterior probability of saccade, under each MDP model. Clearly, in a practical application, one would need to estimate (subject specific) parameters that best accounted for the observed behavior (Schwartenbeck and Friston 2016). However, in this instance, there are no unknown parameters and the log evidence for any given model reduces to the expected log likelihood, under that model. Given that we know each set of lesion data was generated by one of the models, we can calculate the posterior probabilities of each model using a softmax function of the likelihoods for each synthetic dataset.

**Figure 8.**
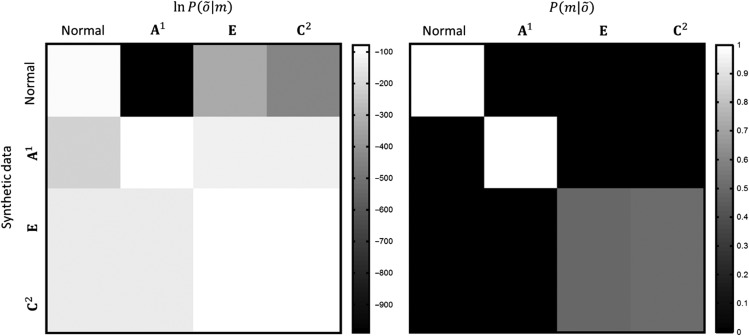
Confusion matrices constructed from 40 saccades. The matrix on the left shows the (log) model evidence $\ln\;P(\tilde{o}|m)$ for each model, m (columns), given synthetic eye tracking data,$\;\tilde{o}$ generated from each model (rows). This is equivalent to the (log) likelihood or model evidence, as there were no unknown parameters. These results were generated using multiscale representations with lesions at the coarsest resolution in all cases. On the right is the matrix of posterior probabilities $P(m|\tilde{o})$. This is obtained from the matrix on the left, using a softmax function applied to the log evidence is in each row (i.e., for different models of each synthetic dataset).

The results of this Bayesian model comparison are shown in Figure 8. It is clear from the confusion matrix shown in the figure that one can reliably disambiguate between health and pathology. Furthermore, the lesions in $A^{1}$ (i.e., a synthetic disconnection between dorsal and ventral attentional systems), although visually very similar to those of the other 2 lesion models, produce a characteristic behavioral pattern, allowing the lesion identity to be recovered. We aim to use this disambiguation to provide an empirical test of our anatomical model. If we use transcranial magnetic stimulation to disrupt the communication between dorsal frontal and right temporoparietal regions, we expect to find that a lesion deficit analysis using eye movements will find greater evidence for an A1 lesion than for any of the other lesion models. Lesions of E and $C^{2}$ could not be disambiguated from one another using only synthetic saccades. That the latter model has a greater posterior probability for both sets of simulated data suggests that this is a simpler explanation for the data, and has incurred a lower complexity penalty during Bayesian model comparison. However, they were clearly identified as being abnormal, and not due to simulated lesions of the superior longitudinal fasciculus; that is, $A^{1}$. This suggests that distinguishing between the 2 may require an additional data modality, such as reaction time, pupillometry, or electrophysiology.

## Theoretical Neurobiology

The work presented in this paper closely relates to a number of recent advances in theoretical neurobiology. We have built upon previous formulations of visual exploration under active inference (Friston, Adams, et al. 2012; Mirza *et al.* 2016), but there are number of important distinctions between these accounts and the current work. The first is that we have used active inference to address the impact of (computational) lesions, and to demonstrate how neuropsychological disorders can be described functionally in terms of pathological priors.

The second difference is subtle: the formulations mentioned above involved the selection of saccadic targets to minimize uncertainty. This is shared with the current work, and with earlier theories of visual salience (Itti and Baldi 2006), but the quantities about which the simulated agent is uncertain differ. Previously, we have emphasized uncertainty about hidden states in the environment. Here, we focus on the uncertainty about the relationship between hidden states and their sensory consequences. It is this important difference that facilitates the analysis of disconnection syndromes, as these can be formulated as disruption of sensorimotor contingencies.

Previous models have addressed attentional processes in general (Bundesen 1998; Heinke and Humphreys 2005), and neglect specifically (Kinsbourne 1970; Heinke and Humphreys 2003). Our approach complements many of these models, while making use of more recent theoretical developments. The belief update scheme we have employed has been used to reproduce a range of other behaviors (FitzGerald et al. 2014; Moutoussis et al. 2014; Friston et al. 2015), physiological responses (Friston et al. 2014; Schwartenbeck, FitzGerald, Mathys, Dolan and Friston 2015), and pathologies (Schwartenbeck, FitzGerald, Mathys, Dolan, Wurst, et al. 2015), emphasizing its plausibility as a description of brain function. Additionally, our use of active inference allows us to appeal to a physiologically plausible process theory (Friston, FitzGerald, Rigoli, Schwartenbeck and Pezzulo 2016), that facilitates the formation of empirical hypotheses about electrophysiological data. For example, we would expect that there would be an increase in the effective connectivity (in healthy subjects) between the regions connected by the second branch of the superior longitudinal fasciculus as Dirichlet parameters are accumulated. We anticipate that this should be reflected in the activities of neurons in these brain regions, and that dynamic causal modeling for evoked responses (David et al. 2006) provides a means to test this hypothesis experimentally. The simulation of eye movements adds to this, as we can use this behavioral data to complement imaging data as in previous experimental work in this area (Adams et al. 2016).

## Conclusion

Visual neglect can be formulated as a computational bias in an active inference scheme that can be quantified in terms of abnormal prior beliefs. In the above, we identified 3, theoretically motivated, functional lesions. On defining a generative MDP model that performed a cancellation task, we found that the connectivity implied by the model structure corresponded well to the anatomy of the dorsal and ventral attention networks, in addition to their subcortical influences. The functional lesions in this anatomical assignment matched lesions associated with visual neglect; namely, in the second branch of the superior longitudinal fasciculus, the putamen, and the pulvinar. The saccadic behavior generated under these lesion models closely resembles that of patients with visual neglect. To provide a more realistic spatial representation, we used a multiscale encoding of visual state space, which implements a multiscale resolution. This allowed us to demonstrate visual neglect at different scales. Encouragingly, although the saccadic behaviors appeared homogenous across each lesion model, we found that we could recover distinct groups of lesions by comparing the evidence for each lesion in synthetic data. In principle, this demonstrates that computational phenotyping of visual neglect patients is possible.

## Appendix

**Update equations**

Using the mean field approximation, and factorizing the joint distribution according to the generative model, the free energy can be rewritten as follows:

$$\begin{array}{cc} F & =E_{Q\left(A|\pi \right)Q\left(\pi \right)\prod_{\tau }Q\left(s_{\tau }|\pi \right)}\;[\ln\;Q\left(A|\pi \right)Q\left(\pi \right)\prod_{\tau }Q\left(s_{\tau }|\pi \right)\\ & \;-\;\ln\;P\left(\pi \right)P\left(A\right)P\left(s_{1}\right)\;P\left(o_{1}|s_{1}\right)\prod_{\tau =2}P\left(s_{\tau }|s_{\tau -1},\pi \right)\;P\;(o_{\tau }|s_{\tau })] \end{array}$$

To find the variational solution to the approximate posterior for $s_{t}|\pi$, we can omit all terms which are constant with respect to this variable. This gives:

$$\begin{array}{cc} F(\pi ) & =E_{Q\left(s_{t}|\pi \right)}\left[\ln\;Q(s_{t}|\pi )\right]-E_{Q\left(A|\pi \right)Q\left(s_{t}|\pi \right)}\left[\ln\;P\left(o_{t}|s_{t},A\right)\right]\\ & \;-E_{Q\left(s_{t-1}|\pi \right)Q\left(s_{t}|\pi \right)}\left[\ln\;P\left(s_{t}|s_{t-1},\pi \right)\right]\\ & \;-E_{Q\left(s_{t}|\pi \right)Q\left(s_{t+1}|\pi \right)}\left[\ln\;P(s_{t+1}|s_{t},\pi )\right] \end{array}$$

Taking the functional derivative with respect to $Q(s_{t}|\pi )$

$$\begin{array}{cc} \frac{\delta F(\pi )}{\delta Q\left(s_{t}|\pi \right)} & =\;\ln\;Q(s_{t}|\pi )-E_{Q\left(A|\pi \right)}\;\left[\ln\;P\left(o_{t}|s_{t},A\right)\right]\\ & \;-E_{Q\left(s_{t-1}|\pi \right)}\left[\ln\;P\left(s_{t}|s_{t-1},\pi \right)\right]-E_{Q\left(s_{t+1}|\pi \right)}\left[\ln\;P(s_{t+1}|s_{t},\pi )\right] \end{array}$$

Setting equal to zero and rearranging gives the variational solution

$$\begin{array}{cc} \ln\;Q(s_{t}|\pi ) & =E_{Q\left(A|\pi \right)}\left[\ln\;P\left(o_{t}|s_{t},A\right)\right]\\ & \;+E_{Q\left(s_{t-1}|\pi \right)}\left[\ln\;P\left(s_{t}|s_{t-1},\pi \right)\right]\\ & \;+E_{Q\left(s_{t+1}|\pi \right)}\left[\ln\;P(s_{t+1}|s_{t},\pi )\right]\\ & \;=\;\ln\;A\;\cdot \;o_{t}+\;\ln\;B_{\pi ,t-1}s_{\pi ,t-1}+\;\ln\;B_{\pi ,t}\;\cdot \;s_{\pi ,t+1} \end{array}$$

This can be used to set up a biologically plausible gradient descent scheme by defining an error term, which expresses the difference between this and the current belief

$$\varepsilon_{\pi ,t}=\;\ln\;A\;\cdot \;o_{t}+\;\ln\;B_{\pi ,t-1}s_{\pi ,t-1}+\;\ln\;B_{\pi ,t}\;\cdot \;s_{\pi ,t+1}-\;\ln\;s_{\pi ,t}$$

When this error is positive, $s_{\pi ,t}$ should increase. The gradient descent on variational free energy then corresponds to an ascent of the form:

$$s_{\pi ,t}=\sigma \left(\nu_{\pi ,t}\right)\;:{\dot{\nu }}_{\pi ,t}=\varepsilon_{\pi ,t}$$

These are the dynamics for the state updates in Figure 2.

## References

[bhx316C1] Adams RA, Bauer M, Pinotsis D, Friston KJ 2016 Dynamic causal modelling of eye movements during pursuit: confirming precision-encoding in V1 using MEG. Neuroimage. 132:175–189. 26921713 10.1016/j.neuroimage.2016.02.055 PMC4862965

[bhx316C2] Albert ML 1973 A simple test of visual neglect. Neurology. 23:658. 4736313 10.1212/wnl.23.6.658

[bhx316C3] Andersen RA, Essick GK, Siegel RM 1985 Encoding of spatial location by posterior parietal neurons. Science. 230:456. 4048942 10.1126/science.4048942

[bhx316C4] Andrade K, Samri D, Sarazin M, de Souza LC, Cohen L, Thiebaut de Schotten M, Dubois B, Bartolomeo P 2010 Visual neglect in posterior cortical atrophy. BMC Neurol. 10:68. 20698982 10.1186/1471-2377-10-68 PMC2924848

[bhx316C5] Auclair L, Siéroff E, Kocer S 2008 A case of spatial neglect dysgraphia in Wilson’s Disease. Arch Clin Neuropsychol. 23:47–62. 17945465 10.1016/j.acn.2007.08.011

[bhx316C6] Bartolomeo P 2014 Spatially biased decisions: toward a dynamic interactive model of visual neglect In: Tracy J, Hampstead B, Sathian K, editors Cognitive plasticity in neurologic disorders. Oxford: Oxford University Press p. 299.

[bhx316C7] Bartolomeo P, Thiebaut de Schotten M, Chica AB 2012 Brain networks of visuospatial attention and their disruption in visual neglect. Front Hum Neurosci. 6:110. 22586384 10.3389/fnhum.2012.00110 PMC3343690

[bhx316C8] Bartolomeo P, Thiebaut de Schotten M, Doricchi F 2007 Left unilateral neglect as a disconnection syndrome. Cereb Cortex. 17:2479–2490. 17272263 10.1093/cercor/bhl181

[bhx316C9] Bays PM, Singh-Curry V, Gorgoraptis N, Driver J, Husain M 2010 Integration of goal- and stimulus-related visual signals revealed by damage to human parietal cortex. J Neurosci. 30:5968. 20427656 10.1523/JNEUROSCI.0997-10.2010 PMC4164540

[bhx316C10] Beal MJ 2003 Variational algorithms for approximate Bayesian inference. United Kingdom: University of London.

[bhx316C11] Behrens TEJ, Johansen-Berg H, Woolrich MW, Smith SM, Wheeler-Kingshott CAM, Boulby PA, Barker GJ, Sillery EL, Sheehan K, Ciccarelli O, et al 2003 Non-invasive mapping of connections between human thalamus and cortex using diffusion imaging. Nat Neurosci. 6:750–757. 12808459 10.1038/nn1075

[bhx316C12] Berson DM, McIlwain JT 1983 Visual cortical inputs to deep layers of cat’s superior colliculus. J Neurophysiol. 50:1143. 6644364 10.1152/jn.1983.50.5.1143

[bhx316C13] Blei DM, Ng AY, Jordan MI 2003 Latent Dirichlet allocation. J Mach Learn Res. 3:993–1022.

[bhx316C14] Bourgeois A, Chica AB, Migliaccio R, Bayle DJ, Duret C, Pradat-Diehl P, Lunven M, Pouget P, Bartolomeo P 2015 Inappropriate rightward saccades after right hemisphere damage: oculomotor analysis and anatomical correlates. Neuropsychologia. 73:1–11. 25930032 10.1016/j.neuropsychologia.2015.04.013

[bhx316C15] Brown TH, Zhao Y, Leung V 2009 Hebbian plasticity A2—squire In: Larry R, editor Encyclopedia of neuroscience. Oxford: Academic Press p. 1049–1056.

[bhx316C16] Bruce CJ, Goldberg ME, Bushnell MC, Stanton GB 1985 Primate frontal eye fields. II. Physiological and anatomical correlates of electrically evoked eye movements. J Neurophysiol. 54:714–734. 4045546 10.1152/jn.1985.54.3.714

[bhx316C17] Bundesen C 1998 A computational theory of visual attention. Philos Trans R Soc London B Biol Sci. 353:1271–1281. 9770221 10.1098/rstb.1998.0282 PMC1692331

[bhx316C18] Conant RC, Ashby WR 1970 Every good regulator of a system must be a model of that system. Int J Syst Sci. 1:89–97.

[bhx316C19] Corbetta M, Akbudak E, Conturo TE, Snyder AZ, Ollinger JM, Drury HA, Linenweber MR, Petersen SE, Raichle ME, Van Essen DC, et al 1998 A common network of functional areas for attention and eye movements. Neuron. 21:761–773. 9808463 10.1016/s0896-6273(00)80593-0

[bhx316C20] Corbetta M, Kincade JM, Ollinger JM, McAvoy MP, Shulman GL 2000 Voluntary orienting is dissociated from target detection in human posterior parietal cortex. Nat Neurosci. 3:292–297. 10700263 10.1038/73009

[bhx316C21] Corbetta M, Kincade JM, Shulman GL 2002 Neural systems for visual orienting and their relationships to spatial working memory. J Cogn Neurosci. 14:508–523. 11970810 10.1162/089892902317362029

[bhx316C22] Corbetta M, Shulman GL 2002 Control of goal-directed and stimulus-driven attention in the brain. Nat Rev Neurosci. 3:201–215. 11994752 10.1038/nrn755

[bhx316C23] Corbetta M, Shulman GL 2011 Spatial neglect and attention networks. Annu Rev Neurosci. 34:569–599. 21692662 10.1146/annurev-neuro-061010-113731 PMC3790661

[bhx316C24] Daunizeau J, den Ouden HEM, Pessiglione M, Kiebel SJ, Stephan KE, Friston KJ 2010 Observing the observer (I): meta-Bayesian models of learning and decision-making. PLoS One. 5:e15554. 21179480 10.1371/journal.pone.0015554 PMC3001878

[bhx316C25] David O, Kiebel SJ, Harrison LM, Mattout J, Kilner JM, Friston KJ 2006 Dynamic causal modeling of evoked responses in EEG and MEG. NeuroImage. 30:1255–1272. 16473023 10.1016/j.neuroimage.2005.10.045

[bhx316C26] Dayan P, Hinton GE, Neal RM, Zemel RS 1995 The Helmholtz machine. Neural Comput. 7:889–904. 7584891 10.1162/neco.1995.7.5.889

[bhx316C27] Di Stefano F, Floris G, Vacca M, Serra G, Cannas A, Borghero G, Marrosu MG, Marrosu F 2013 Transient unilateral spatial neglect during aura in a woman with sporadic hemiplegic migraine. Cephalalgia. 33:1194–1197. 23674829 10.1177/0333102413487446

[bhx316C28] Dietz MJ, Friston KJ, Mattingley JB, Roepstorff A, Garrido MI 2014 Effective connectivity reveals right-hemisphere dominance in audiospatial perception: implications for models of spatial neglect. J Neurosci. 34:5003–5011. 24695717 10.1523/JNEUROSCI.3765-13.2014 PMC3972725

[bhx316C29] Doricchi F, Tomaiuolo F 2003 The anatomy of neglect without hemianopia: a key role for parietal–frontal disconnection? Neuroreport. 14:2239–2243. 14625455 10.1097/00001756-200312020-00021

[bhx316C30] Dosenbach NUF, Visscher KM, Palmer ED, Miezin FM, Wenger KK, Kang HC, Burgund ED, Grimes AL, Schlaggar BL, Petersen SE 2006 A Core System for the implementation of task sets. Neuron. 50:799–812. 16731517 10.1016/j.neuron.2006.04.031 PMC3621133

[bhx316C31] Driver J, Baylis GC, Goodrich SJ, Rafal RD 1994 Axis-based neglect of visual shapes. Neuropsychologia. 32:1353–1356. 7877744 10.1016/0028-3932(94)00068-9

[bhx316C32] Driver J, Pouget A 2000 Object-centered visual neglect, or relative egocentric neglect? J Cogn Neurosci. 12:542–545. 10931777 10.1162/089892900562192

[bhx316C33] Duncan J 2001 An adaptive coding model of neural function in prefrontal cortex. Nat Rev Neurosci. 2:820–829. 11715058 10.1038/35097575

[bhx316C34] Ferber S, Karnath H-O 2001 How to assess spatial neglect-line bisection or cancellation tasks? J Clin Exp Neuropsychol. 23:599–607. 11778637 10.1076/jcen.23.5.599.1243

[bhx316C35] FitzGerald T, Dolan R, Friston K 2014 Model averaging, optimal inference, and habit formation. Front Hum Neurosci. doi:10.3389/fnhum.2014.00457. 10.3389/fnhum.2014.00457 PMC4071291 25018724

[bhx316C36] Fries W 1984 Cortical projections to the superior colliculus in the macaque monkey: a retrograde study using horseradish peroxidase. J Comp Neurol. 230:55–76. 6096414 10.1002/cne.902300106

[bhx316C37] Fries W 1985 Inputs from motor and premotor cortex to the superior colliculus of the macaque monkey. Behav Brain Res. 18:95–105. 3913446 10.1016/0166-4328(85)90066-x

[bhx316C38] Friston K 2003 Learning and inference in the brain. Neural Netw. 16:1325–1352. 14622888 10.1016/j.neunet.2003.06.005

[bhx316C39] Friston K 2009 The free-energy principle: a rough guide to the brain? Trends Cogn Sci. 13:293–301. 19559644 10.1016/j.tics.2009.04.005

[bhx316C40] Friston K, Adams RA, Perrinet L, Breakspear M 2012 Perceptions as hypotheses: saccades as experiments. Front Psychol. 3:151. 22654776 10.3389/fpsyg.2012.00151 PMC3361132

[bhx316C41] Friston K, FitzGerald T, Rigoli F, Schwartenbeck P, O’Doherty J, Pezzulo G 2016 Active inference and learning. Neurosci Biobehav Rev. 68:862–879. 27375276 10.1016/j.neubiorev.2016.06.022 PMC5167251

[bhx316C42] Friston K, FitzGerald T, Rigoli F, Schwartenbeck P, Pezzulo G 2016 Active inference: a process theory. Neural Comput. 29:1–49. 27870614 10.1162/NECO_a_00912

[bhx316C43] Friston K, Kilner J, Harrison L 2006 A free energy principle for the brain. J Physiol Paris. 100:70–87. 17097864 10.1016/j.jphysparis.2006.10.001

[bhx316C44] Friston K, Rigoli F, Ognibene D, Mathys C, Fitzgerald T, Pezzulo G 2015 Active inference and epistemic value. Cogn Neurosci. 6:187–214. 25689102 10.1080/17588928.2015.1020053

[bhx316C45] Friston K, Samothrakis S, Montague R 2012 Active inference and agency: optimal control without cost functions. Biol Cybern. 106:523–541. 22864468 10.1007/s00422-012-0512-8

[bhx316C46] Friston K, Schwartenbeck P, Fitzgerald T, Moutoussis M, Behrens T, Dolan R 2013 The anatomy of choice: active inference and agency. Front Hum Neurosci. 7:598. 10.3389/fnhum.2013.00598 PMC3782702 24093015

[bhx316C47] Friston K, Schwartenbeck P, FitzGerald T, Moutoussis M, Behrens T, Dolan RJ 2014 The anatomy of choice: dopamine and decision-making. Philos Trans R Soc B Biol Sci. 369:20130481. 10.1098/rstb.2013.0481 PMC4186234 25267823

[bhx316C48] Friston KJ, Rosch R, Parr T, Price C, Bowman H 2017 Deep temporal models and active inference. Neurosci Biobehav Rev. 77:388–402. 28416414 10.1016/j.neubiorev.2017.04.009 PMC5461873

[bhx316C49] Fruhmann Berger M, Johannsen L, Karnath H-O 2008 Time course of eye and head deviation in spatial neglect. Neuropsychology. 22:697–702. 18999342 10.1037/a0013351

[bhx316C50] Fullerton KJ, McSherry D, Stout RW 1986 Albert’s test: a neglected test of perceptual neglect. Lancet. 327:430–432. 10.1016/s0140-6736(86)92381-0 2868349

[bhx316C51] Gaymard B, Lynch J, Ploner CJ, Condy C, Rivaud-Péchoux S 2003 The parieto-collicular pathway: anatomical location and contribution to saccade generation. Eur J Neurosci. 17:1518–1526. 12713655 10.1046/j.1460-9568.2003.02570.x

[bhx316C52] Gertler TS, Chan CS, Surmeier DJ 2008 Dichotomous anatomical properties of adult striatal medium spiny neurons. J Neurosci. 28:10814. 18945889 10.1523/JNEUROSCI.2660-08.2008 PMC3235748

[bhx316C53] Gilad R, Sadeh M, Boaz M, Lampl Y 2006 Visual spatial neglect in multiple sclerosis. Cortex. 42:1138–1142. 17209419 10.1016/s0010-9452(08)70226-0

[bhx316C54] Goodale MA, Milner AD 1992 Separate visual pathways for perception and action. Trends Neurosci. 15:20–25. 1374953 10.1016/0166-2236(92)90344-8

[bhx316C55] Grimsen C, Hildebrandt H, Fahle M 2008 Dissociation of egocentric and allocentric coding of space in visual search after right middle cerebral artery stroke. Neuropsychologia. 46:902–914. 18206963 10.1016/j.neuropsychologia.2007.11.028

[bhx316C56] Haber SN 2003 The primate basal ganglia: parallel and integrative networks. J Chem Neuroanat. 26:317–330. 14729134 10.1016/j.jchemneu.2003.10.003

[bhx316C57] Halligan PW, Marshall JC 1998 Neglect of awareness. Conscious Cogn. 7:356–380. 9787050 10.1006/ccog.1998.0362

[bhx316C58] He BJ, Snyder AZ, Vincent JL, Epstein A, Shulman GL, Corbetta M 2007 Breakdown of functional connectivity in frontoparietal networks underlies behavioral deficits in spatial neglect. Neuron. 53:905–918. 17359924 10.1016/j.neuron.2007.02.013

[bhx316C59] Hebb DO 1949 The first stage of perception: growth of the assembly. The Organization of Behavior: pp. 60–78.

[bhx316C60] Heilman KM, Howell GJ 1980 Seizure-induced neglect. J Neurol Neurosurg Psychiatry. 43:1035–1040. 6777464 10.1136/jnnp.43.11.1035 PMC490757

[bhx316C61] Heinke D, Humphreys GW 2003 Attention, spatial representation, and visual neglect: simulating emergent attention and spatial memory in the selective attention for identification model (SAIM). Psychol Rev. 110:29–87. 12529057 10.1037/0033-295x.110.1.29

[bhx316C62] Heinke D, Humphreys GW 2005 Computational models of visual selective attention: a review. Connect Models Cogn Psychol. 1:273–312.

[bhx316C63] Hikosaka O, Wurtz RH 1983 Visual and oculomotor functions of monkey substantia nigra pars reticulata. IV. Relation of substantia nigra to superior colliculus. J Neurophysiol. 49:1285. 6306173 10.1152/jn.1983.49.5.1285

[bhx316C64] Hikosaka O, Wurtz RH 1985a. Modification of saccadic eye movements by GABA-related substances. I. Effect of muscimol and bicuculline in monkey superior colliculus. J Neurophysiol. 53:266. 2983037 10.1152/jn.1985.53.1.266

[bhx316C65] Hillis AE, Newhart M, Heidler J, Barker PB, Herskovits EH, Degaonkar M 2005 Anatomy of spatial attention: insights from perfusion imaging and hemispatial neglect in acute stroke. J Neurosci. 25:3161. 15788773 10.1523/JNEUROSCI.4468-04.2005 PMC6725074

[bhx316C66] Ho AK, Manly T, Nestor PJ, Sahakian BJ, Bak TH, Robbins TW, Rosser AE, Barker RA 2003 A case of unilateral neglect in Huntington’s disease. Neurocase. 9:261–273. 12925932 10.1076/neur.9.3.261.15559

[bhx316C67] Hubel DH, Livingstone MS 1987 Segregation of form, color, and stereopsis in primate area 18. J Neurosci. 7:3378. 2824714 10.1523/JNEUROSCI.07-11-03378.1987 PMC6569042

[bhx316C68] Husain M, Mannan S, Hodgson T, Wojciulik E, Driver J, Kennard C 2001 Impaired spatial working memory across saccades contributes to abnormal search in parietal neglect. Brain. 124:941–952. 11335696 10.1093/brain/124.5.941

[bhx316C69] Itti L, Baldi P 2006 Bayesian surprise attracts human attention. Adv Neural Inf Process Syst. 18:547. 10.1016/j.visres.2008.09.007 PMC2782645 18834898

[bhx316C70] Jahanshahi M, Obeso I, Rothwell JC, Obeso JA 2015 A fronto-striato-subthalamic-pallidal network for goal-directed and habitual inhibition. Nat Rev Neurosci. 16:719–732. 26530468 10.1038/nrn4038

[bhx316C71] Kanai R, Komura Y, Shipp S, Friston K 2015 Cerebral hierarchies: predictive processing, precision and the pulvinar. Philos Trans R Soc B Biol Sci. 370:1668. 10.1098/rstb.2014.0169 PMC4387510 25823866

[bhx316C72] Karnath H-O, Rorden C 2012 The anatomy of spatial neglect. Neuropsychologia. 50:1010–1017. 21756924 10.1016/j.neuropsychologia.2011.06.027 PMC3348466

[bhx316C73] Karnath HO, Himmelbach M, Rorden C 2002 The subcortical anatomy of human spatial neglect: putamen, caudate nucleus and pulvinar. Brain. 125:350–360. 11844735 10.1093/brain/awf032

[bhx316C74] Kato M, Miyashita N, Hikosaka O, Matsumura M, Usui S, Kori A 1995 Eye movements in monkeys with local dopamine depletion in the caudate nucleus. I. Deficits in spontaneous saccades. J Neurosci. 15:912. 7823189 10.1523/JNEUROSCI.15-01-00912.1995 PMC6578295

[bhx316C75] Kinsbourne M 1970 A model for the mechanism of unilateral neglect of space. Trans Am Neurol Assoc. 95:143–146. 5514359

[bhx316C76] Kirshner HS 2003 Chapter 140—speech and language disorders A2—Samuels, Martin A In: Feske SK, editor Office practice of neurology. 2nd ed Philadelphia: Churchill Livingstone p. 890–895.

[bhx316C77] Kori A, Miyashita N, Kato M, Hikosaka O, Usui S, Matsumura M 1995 Eye movements in monkeys with local dopamine depletion in the caudate nucleus. II. Deficits in voluntary saccades. J Neurosci. 15:928. 7823190 10.1523/JNEUROSCI.15-01-00928.1995 PMC6578280

[bhx316C78] Künzle H, Akert K 1977 Efferent connections of cortical, area 8 (frontal eye field) in Macaca fascicularis. A reinvestigation using the autoradiographic technique. J Comp Neurol. 173:147–163. 403205 10.1002/cne.901730108

[bhx316C79] Ladavas E, Zeloni G, Zaccara G, Gangemi P 1997 Eye movements and orienting of attention in patients with visual neglect. J Cogn Neurosci. 9:67–74. 23968180 10.1162/jocn.1997.9.1.67

[bhx316C80] Li D, Karnath H-O, Rorden C 2014 Egocentric representations of space co-exist with allocentric representations: evidence from spatial neglect. Cortex. 58:161–169. 25038308 10.1016/j.cortex.2014.06.012 PMC4130897

[bhx316C81] Livingstone M, Hubel D 1988 Segregation of form, color, movement, and depth: anatomy, physiology, and perception. Science. 240:740. 3283936 10.1126/science.3283936

[bhx316C82] Makris N, Kennedy DN, McInerney S, Sorensen AG, Wang R, Caviness JVS, Pandya DN 2004 Segmentation of subcomponents within the superior longitudinal fascicle in humans: a quantitative, in vivo, DT-MRI study. Cereb Cortex. 15:854–869. 15590909 10.1093/cercor/bhh186

[bhx316C83] Medina J, Kannan V, Pawlak MA, Kleinman JT, Newhart M, Davis C, Heidler-Gary JE, Herskovits EH, Hillis AE 2009 Neural substrates of visuospatial processing in distinct reference frames: evidence from unilateral spatial neglect. J Cogn Neurosci. 21:2073–2084. 19016599 10.1162/jocn.2008.21160 PMC2828044

[bhx316C84] Mirza MB, Adams RA, Mathys CD, Friston KJ 2016 Scene construction, visual foraging, and active inference. Front Comput Neurosci. 10:56. 27378899 10.3389/fncom.2016.00056 PMC4906014

[bhx316C85] Mongillo G, Barak O, Tsodyks M 2008 Synaptic theory of working memory. Science. 319:1543–1546. 18339943 10.1126/science.1150769

[bhx316C86] Moutoussis M, Trujillo-Barreto NJ, El-Deredy W, Dolan RJ, Friston KJ 2014 A formal model of interpersonal inference. Front Hum Neurosci. 8:160. 24723872 10.3389/fnhum.2014.00160 PMC3971175

[bhx316C87] Nealey TA, Maunsell JH 1994 Magnocellular and parvocellular contributions to the responses of neurons in macaque striate cortex. J Neurosci. 14:2069. 8158257 10.1523/JNEUROSCI.14-04-02069.1994 PMC6577134

[bhx316C88] Ota H, Fujii T, Suzuki K, Fukatsu R, Yamadori A 2001 Dissociation of body-centered and stimulus-centered representations in unilateral neglect. Neurology. 57:2064–2069. 11739827 10.1212/wnl.57.11.2064

[bhx316C89] Parr T, Friston KJ 2017 The active construction of the visual world. Neuropsychologia. 104:92–101. 28782543 10.1016/j.neuropsychologia.2017.08.003 PMC5637165

[bhx316C90] Petersen SE, Robinson DL, Keys W 1985 Pulvinar nuclei of the behaving rhesus monkey: visual responses and their modulation. J Neurophysiol. 54:867. 4067625 10.1152/jn.1985.54.4.867

[bhx316C91] Pouget A, Sejnowski TJ 1997 A new view of hemineglect based on the response properties of parietal neurones. Philos Trans R Soc B Biol Sci. 352:1449–1459. 10.1098/rstb.1997.0131 PMC1692050 9368933

[bhx316C92] Pouget A, Sejnowski TJ 2001 Simulating a lesion in a basis function model of spatial representations: comparison with hemineglect. Psychol Rev. 108:653. 11488381 10.1037/0033-295x.108.3.653

[bhx316C93] Ptak R, Schnider A 2010 The dorsal attention network mediates orienting toward behaviorally relevant stimuli in spatial neglect. J Neurosci. 30:12557. 20861361 10.1523/JNEUROSCI.2722-10.2010 PMC6633576

[bhx316C94] Raybourn MS, Keller EL 1977 Colliculoreticular organization in primate oculomotor system. J Neurophysiol. 40:861. 407334 10.1152/jn.1977.40.4.861

[bhx316C95] Robinson DL, Petersen SE 1992 The pulvinar and visual salience. Trends Neurosci. 15:127–132. 1374970 10.1016/0166-2236(92)90354-b

[bhx316C96] Rorden C, Hjaltason H, Fillmore P, Fridriksson J, Kjartansson O, Magnusdottir S, Karnath H-O 2012 Allocentric neglect strongly associated with egocentric neglect. Neuropsychologia. 50:1151–1157. 22608082 10.1016/j.neuropsychologia.2012.03.031 PMC3358702

[bhx316C97] Rushmore RJ, Valero-Cabre A, Lomber SG, Hilgetag CC, Payne BR 2006 Functional circuitry underlying visual neglect. Brain. 129:1803–1821. 16731540 10.1093/brain/awl140

[bhx316C98] Rushworth MFS, Behrens TEJ, Johansen-Berg H 2005 Connection patterns distinguish 3 regions of human parietal cortex. Cereb Cortex. 16:1418–1430. 16306320 10.1093/cercor/bhj079

[bhx316C99] Sajad A, Sadeh M, Keith GP, Yan X, Wang H, Crawford JD 2015 Visual–motor transformations within frontal eye fields during head-unrestrained gaze shifts in the monkey. Cereb Cortex. 25:3932–3952. 25491118 10.1093/cercor/bhu279 PMC4585524

[bhx316C100] Schiller PH, Sandell JH, Maunsell JH 1987 The effect of frontal eye field and superior colliculus lesions on saccadic latencies in the rhesus monkey. J Neurophysiol. 57:1033. 3585453 10.1152/jn.1987.57.4.1033

[bhx316C101] Schiller PH, True SD, Conway JL 1980 Deficits in eye movements following frontal eye-field and superior colliculus ablations. J Neurophysiol. 44:1175. 6778974 10.1152/jn.1980.44.6.1175

[bhx316C102] Schomer AC, Drislane FW 2015 Severe hemispatial neglect as a manifestation of seizures and nonconvulsive status epilepticus: utility of prolonged EEG monitoring. J Clin Neurophysiol. 32:e4–e7. 25830272 10.1097/WNP.0000000000000107

[bhx316C103] Schwartenbeck P, FitzGerald TH, Mathys C, Dolan R, Friston K 2015 The dopaminergic midbrain encodes the expected certainty about desired outcomes. Cereb Cortex. 25:3434–3445. 25056572 10.1093/cercor/bhu159 PMC4585497

[bhx316C104] Schwartenbeck P, FitzGerald TH, Mathys C, Dolan R, Wurst F, Kronbichler M, Friston K 2015 Optimal inference with suboptimal models: addiction and active Bayesian inference. Med Hypotheses. 84:109–117. 25561321 10.1016/j.mehy.2014.12.007 PMC4312353

[bhx316C105] Schwartenbeck P, Friston K 2016 Computational phenotyping in psychiatry: a worked example. eNeuro. 3:ENEURO.0049-0016.2016. 10.1523/ENEURO.0049-16.2016 PMC4969668 27517087

[bhx316C106] Shipp S 2003 The functional logic of cortico-pulvinar connections. Philos Trans R Soc B Biol Sci. 358:1605–1624. 10.1098/rstb.2002.1213 PMC1693262 14561322

[bhx316C107] Shipp S 2004 The brain circuitry of attention. Trends Cogn Sci. 8:223–230. 15120681 10.1016/j.tics.2004.03.004

[bhx316C108] Shomstein S, Lee J, Behrmann M 2010 Top-down and bottom-up attentional guidance: investigating the role of the dorsal and ventral parietal cortices. Exp Brain Res. 206:197–208. 20571784 10.1007/s00221-010-2326-z PMC5728384

[bhx316C109] Shook BL, Schlag-Rey M, Schlag J 1990 Primate supplementary eye field: I. Comparative aspects of mesencephalic and pontine connections. J Comp Neurol. 301:618–642. 2273101 10.1002/cne.903010410

[bhx316C110] Shulman GL, Astafiev SV, Franke D, Pope DLW, Snyder AZ, McAvoy MP, Corbetta M 2009 Interaction of stimulus-driven reorienting and expectation in ventral and dorsal fronto-parietal and basal ganglia-cortical networks. J Neurosci. 29:4392–4407. 19357267 10.1523/JNEUROSCI.5609-08.2009 PMC2743562

[bhx316C111] Stevens MC, Calhoun VD, Kiehl KA 2005 Hemispheric differences in hemodynamics elicited by auditory oddball stimuli. NeuroImage. 26:782–792. 15955488 10.1016/j.neuroimage.2005.02.044 PMC2759643

[bhx316C112] Thiebaut de Schotten M, Dell’Acqua F, Forkel SJ, Simmons A, Vergani F, Murphy DGM, Catani M 2011 A lateralized brain network for visuospatial attention. Nat Neurosci. 14:1245–1246. 21926985 10.1038/nn.2905

[bhx316C113] Thiebaut de Schotten M, Urbanski M, Duffau H, Volle E, Lévy R, Dubois B, Bartolomeo P 2005 Direct evidence for a parietal-frontal pathway subserving spatial awareness in humans. Science. 309:2226. 16195465 10.1126/science.1116251

[bhx316C114] Turtzo LC, Kleinman JT, Llinas RH 2008 Capgras syndrome and unilateral spatial neglect in nonconvulsive status epilepticus. Behav Neurol. 20:61–64. 19491475 10.3233/BEN-2008-0210 PMC5452452

[bhx316C115] Ungerleider LG, Christensen CA 1979 Pulvinar lesions in monkeys produce abnormal scanning of a complex visual array. Neuropsychologia. 17:493–501. 117393 10.1016/0028-3932(79)90056-3

[bhx316C116] Ungerleider LG, Haxby JV 1994 ‘What’ and ‘where’ in the human brain. Curr Opin Neurobiol. 4:157–165. 8038571 10.1016/0959-4388(94)90066-3

[bhx316C117] Veale R, Hafed ZM, Yoshida M 2017 How is visual salience computed in the brain? Insights from behaviour, neurobiology and modelling. Philos Trans R Soc B Biol Sci. 372:1714. 10.1098/rstb.2016.0113 PMC5206280 28044023

[bhx316C118] Verdon V, Schwartz S, Lovblad K-O, Hauert C-A, Vuilleumier P 2009 Neuroanatomy of hemispatial neglect and its functional components: a study using voxel-based lesion-symptom mapping. Brain. 133:880–894. 20028714 10.1093/brain/awp305

[bhx316C119] Vossel S, Weidner R, Driver J, Friston KJ, Fink GR 2012 Deconstructing the architecture of dorsal and ventral attention systems with dynamic causal modeling. J Neurosci. 32:10637. 22855813 10.1523/JNEUROSCI.0414-12.2012 PMC3432566

[bhx316C120] Vuilleumier P, Hester D, Assal G, Regli F 1996 Unilateral spatial neglect recovery after sequential strokes. Neurology. 46:184–189. 8559371 10.1212/wnl.46.1.184

[bhx316C121] Wald A 1947 An essentially complete class of admissible decision functions. Ann Math Stat. 18:549–555.

[bhx316C122] Warrington EK, James M 1967 Disorders of visual perception in patients with localised cerebral lesions. Neuropsychologia. 5:253–266.

[bhx316C123] Warrington EK, James M 1988 Visual apperceptive agnosia: a clinico-anatomical study of three cases. Cortex. 24:13–32. 3371008 10.1016/s0010-9452(88)80014-5

[bhx316C124] Warrington EK, Taylor AM 1973 The contribution of the right parietal lobe to object recognition. Cortex. 9:152–164. 4795556 10.1016/s0010-9452(73)80024-3

[bhx316C125] Weller RE, Steele GE, Kaas JH 2002 Pulvinar and other subcortical connections of dorsolateral visual cortex in monkeys. J Comp Neurol. 450:215–240. 12209852 10.1002/cne.10298

[bhx316C126] Yin HH, Knowlton BJ 2006 The role of the basal ganglia in habit formation. Nat Rev Neurosci. 7:464–476. 16715055 10.1038/nrn1919

[bhx316C127] Yue Y, Song W, Huo S, Wang M 2012 Study on the occurrence and neural bases of hemispatial neglect with different reference frames. Arch Phys Med Rehabil. 93:156–162. 22200396 10.1016/j.apmr.2011.07.192

[bhx316C128] Zeki S, Shipp S 1988 The functional logic of cortical connections. Nature. 335:311–317. 3047584 10.1038/335311a0

[bhx316C129] Zeki S, Shipp S 1989 Modular connections between areas V2 and V4 of macaque monkey visual cortex. Eur J Neurosci. 1:494–506. 12106135 10.1111/j.1460-9568.1989.tb00356.x

